# Systematic exploration of autonomous modules in noisy microRNA-target networks for testing the generality of the ceRNA hypothesis

**DOI:** 10.1186/1471-2164-15-1178

**Published:** 2014-12-24

**Authors:** Danny Kit-Sang Yip, Iris K Pang, Kevin Y Yip

**Affiliations:** Department of Computer Science and Engineering, The Chinese University of Hong Kong, Shatin, New Territories, Hong Kong; School of Life Sciences, The Chinese University of Hong Kong, Shatin, New Territories, Hong Kong; Hong Kong Bioinformatics Centre, The Chinese University of Hong Kong, Shatin, New Territories, Hong Kong; CUHK-BGI Innovation Institute of Trans-omics, The Chinese University of Hong Kong, Shatin, New Territories, Hong Kong

**Keywords:** Competing endogeneous RNA, MicroRNA-target bicluster, MicroRNA network

## Abstract

**Background:**

In the competing endogenous RNA (ceRNA) hypothesis, different transcripts communicate through a competition for their common targeting microRNAs (miRNAs). Individual examples have clearly shown the functional importance of ceRNA in gene regulation and cancer biology. It remains unclear to what extent gene expression levels are regulated by ceRNA in general. One major hurdle to studying this problem is the intertwined connections in miRNA-target networks, which makes it difficult to isolate the effects of individual miRNAs.

**Results:**

Here we propose computational methods for decomposing a complex miRNA-target network into largely autonomous modules called microRNA-target biclusters (MTBs). Each MTB contains a relatively small number of densely connected miRNAs and mRNAs with few connections to other miRNAs and mRNAs. Each MTB can thus be individually analyzed with minimal crosstalk with other MTBs. Our approach differs from previous methods for finding modules in miRNA-target networks by not making any pre-assumptions about expression patterns, thereby providing objective information for testing the ceRNA hypothesis. We show that the expression levels of miRNAs and mRNAs in an MTB are significantly more anti-correlated than random miRNA-mRNA pairs and other validated and predicted miRNA-target pairs, demonstrating the biological relevance of MTBs. We further show that there is widespread correlation of expression between mRNAs in same MTBs under a wide variety of parameter settings, and the correlation remains even when co-regulatory effects are controlled for, which suggests potential widespread expression buffering between these mRNAs, which is consistent with the ceRNA hypothesis. Lastly, we also propose a potential use of MTBs in functional annotation of miRNAs.

**Conclusions:**

MTBs can be used to help identify autonomous miRNA-target modules for testing the generality of the ceRNA hypothesis experimentally. The identified modules can also be used to test other properties of miRNA-target networks in general.

**Electronic supplementary material:**

The online version of this article (doi:10.1186/1471-2164-15-1178) contains supplementary material, which is available to authorized users.

## Background

MicroRNAs (miRNAs) are short endogenous RNAs that bind specific sites of messenger RNA (mRNA) targets called miRNA response elements (MREs) with partial or full sequence complementarity. The protein levels of the targets are regulated by the miRNAs through the promotion of RNA degradation or translational repression [1–4]. Based on the distribution of MREs on different mRNAs, one miRNA could target multiple mRNAs, and multiple miRNAs could target the same mRNA, leading to a complex network of miRNA-mRNA interactions [5, 6].

While conventionally miRNAs are considered to regulate their mRNA targets, in theory mRNAs could also back-regulate their targeting miRNAs by affecting their availability in binding other mRNAs [7, 8]. If the expression level of an mRNA is increased, more copies of its targeting miRNAs will bind to it and become less available for binding other targets. These other targets will be de-repressed and their expression levels will increase. Similarly, if the expression level of an mRNA is decreased, more copies of its targeting miRNAs will become available. They will bind more to other targets and will decrease their expression levels. As a result, different targets of a miRNA can buffer each other [9, 10] and display a positive correlation of their expression levels [8]. In general, different transcripts (mRNAs and other non-coding RNAs) with MREs of the same miRNA may compete for the finite copies of the miRNA in a cell. This back-regulation mechanism and its *in vivo* functional roles have been coined the competing endogenous RNA (ceRNA) hypothesis [8].

One interesting example that supports the ceRNA hypothesis was found between the tumor suppressor gene PTEN and its pseudogene PTENP1 [11]. The MREs of some miRNAs that target PTEN, including miR-19b and miR-20a, are preserved in the truncated 3’ end of the PTENP1 transcript, which allow it to act as a miRNA target decoy for PTEN. Indeed, the expression of both PTEN and PTENP1 was repressed by miR-19b and miR-20a in DU145 prostate cancer cells, and their expression levels exhibited a positive correlation across a large number of normal human tissues and prostate tumor samples. Functionally, PTENP1 was found to have tumor suppressive activity and was selectively lost in human cancer, which suggest a potential role of this pseudogene in the normal functioning of PTEN in tumor suppression. Additional evidence of the functional roles of ceRNA in human cancer was reported in the same study and a series of other studies [11–15]. Regulatory interactions between mRNAs that share common MREs had also been discovered in plants, a phenomenon known as “target mimicry” [7, 16].

At a more global scale, the idea that miRNA targets buffer each other has been used by a number of methods to study miRNA-target interactions. Some methods identify the subset of computationally predicted miRNA targets with a positive correlation of expression as the more reliable targets [17–19]. Some methods identify “sponge” modulators of miRNA-target interactions, which are RNAs whose expression is associated with changes in the mutual information between miRNAs and their targets [14]. All these methods assume a certain degree of generality of the ceRNA hypothesis, and require some high-throughput expression data as input.

In contrast, a transcriptome-wide systematic test of the ceRNA hypothesis has been lacking. It has not been certain whether the buffering between miRNA targets is sufficiently strong to be reflected by their expression levels in general. Conceptually, this can be tested by a two-step procedure, namely (1) gathering a list of miRNA-target pairs obtained by a method not considering their expression patterns, and (2) evaluating whether mRNAs targeted by same miRNAs are significantly more correlated in expression than other mRNAs (with proper control for effects due to co-regulation, as discussed in detail below). While conceptually simple, there are a number of issues that make this kind of analysis practically difficult:

1. Noisy miRNA-target networks: Current computational methods for miRNA target prediction have limited accuracy and consistency [20], while the number of experimentally validated miRNA-target pairs is small [21]. False positives and false negatives in the miRNA target predictions would make it difficult to identify mRNAs with common targeting miRNAs.
2. Unshared targeting miRNAs: mRNAs targeted by a common miRNA may individually be targeted by other unshared miRNAs, which could affect their expression levels separately and lower their correlation.
3. Unshared mRNA targets: miRNAs that target a common set of mRNAs may individually have additional unshared targets, which could dilute the buffering effect of their common set of mRNA targets.
4. Partial effects at transcriptional level: The functional effects of miRNAs on their targets are only partially reflected by mRNA levels, while data about protein abundance are not as widely available.
5. Other gene regulatory mechanisms: Gene expression is regulated by a complex system that involves many other components. Even if two mRNAs are competing for their common targeting miRNAs, their expression levels may not appear correlated if they are individually affected by some other regulatory mechanisms. In addition, a miRNA may affect the expression level of a gene indirectly through its targets that directly or indirectly regulate the gene, leading to expression patterns more difficult to analyze.

In this study, we propose computational methods for studying noisy miRNA-target networks that can overcome the first three issues and tolerate the last two. The main idea is to identify small modules in the networks, which we call microRNA-target biclusters (MTBs), without using any expression data as input. It is a novel concept inspired by the related work on biclustering in the literature of gene expression data analysis [22]. Each MTB consists of a set of miRNAs and a set of mRNAs, where (1) the miRNAs target most of the mRNAs in the MTB but few other mRNAs and (2) the mRNAs are targeted by most of the miRNAs in the MTB but few other miRNAs. Each MTB represents a network module that potentially maintains a largely autonomous regulation sub-system. By tuning the level of interactions linking members of an MTB to non-members, and the level of missing intra-MTB interactions allowed, the impacts of false positives and false negatives in the interaction networks on the MTBs (issue 1) and the degree of independence of each MTB (issues 2 and 3) can be controlled.

To show the biological relevance of MTBs, we analyzed the expression patterns of the miRNAs and mRNAs in our MTBs using RNA-seq data from matched cell lines produced by the ENCODE consortium [23, 24]. We show that the MTBs identified by our methods contain miRNAs more anti-correlated in expression with the mRNAs in the same MTBs than both their other targets and random mRNA sets. As proposed in a series of previous studies, this strong anti-correlation observed indicates that the mRNAs in our MTBs are likely true targets of the miRNAs in the same MTBs [25–32]. By using formal validation procedures and considering many different experimental settings, we show that our results are statistically significant and robust. These results suggest that despite the incomplete reflection of the effects of miRNAs at the transcription level (issue 4) and the presence of other transcriptional regulatory mechanisms (issue 5), it is still possible to systematically analyze the effects of miRNAs on their targets using RNA-seq data.

Correspondingly, we observed stronger expression correlations among mRNAs in the same MTBs even if subtle effects due to co-regulation are controlled for. We also found that mRNAs and miRNAs in the same MTBs have related biological functions. Overall, our results suggest widespread expression buffering between mRNAs commonly targeted by the same miRNAs, which is in line with the ceRNA hypothesis.

In the literature of computational analysis of miRNAs, the two main focuses have long been on identifying miRNA-encoding regions from genomes [33–36] and on predicting the targets of individual miRNAs [37, 38]. In line with the latest trend of studying the inter-related miRNA-target interactions from a network perspective [27, 39–44], our work introduces a new way to study these miRNA-target networks by decomposing complex networks into simple modules that can be more easily analyzed.

## Results and discussion

### Defining MTBs and identifying them from miRNA-target networks

**Figure 1:**
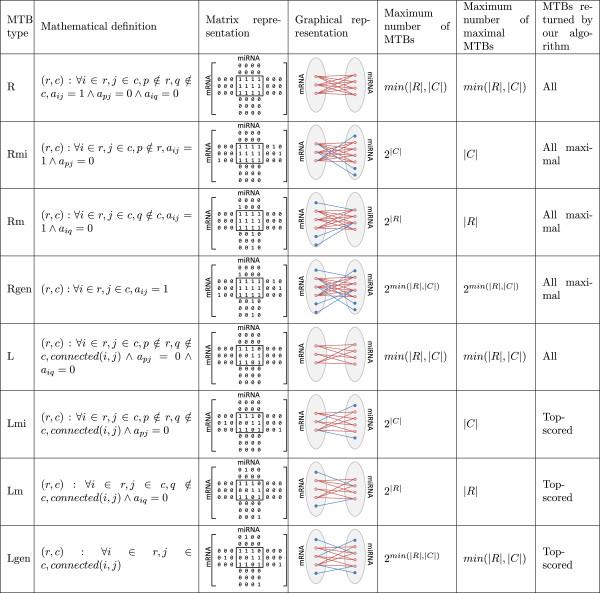
**Summary on the eight types of MTB.** The different types are named according to (1) whether the defining submatrix of an MTB can only contain 1’s (restrictive, R) or is allowed to contain 0’s (loose, L), and (2) whether the miRNAs (mi) are not allowed to have extra targets, the mRNAs (m) are not allowed to be targeted by extra miRNAs, or the general case (gen) that both are allowed. In the formal mathematical definitions, *r* and *c* correspond to the sets of row and column indices defining an MTB, where each row corresponds to an mRNA and each column corresponds to a miRNA. *a*_*ij*_ is the value at row *i* and column *j* of the adjacency matrix. In the matrix representation, an example is shown for each type of MTB, where the sub-matrix enclosed by the rectangle corresponds to the example MTB. For visualization purpose, they are drawn to occupy consecutive rows and columns, but this is not required in the actual definitions of the MTB types. Values of irrelevant cells, i.e., those not on the rows and columns defining the MTB, are omitted. In the graphical representation, the red nodes are the mRNAs and miRNAs defining the example MTB, red lines are edges between them, and blue lines are edges connecting them to mRNAs or miRNAs outside the MTB. In the formulas for showing the number of MTBs of each type, *R* and *C* are the full sets of miRNAs and genes in the miRNA-target network, respectively, and |*R*| and |*C*| are their sizes. The function *connected*(*i*,*j*) means the nodes in the graphical representation corresponding to row *i* and column *j* are connected, which can be formally defined as .

We collected computationally predicted human miRNA targets from 5 prediction methods. We combined these predictions to form a high-confidence set and a high-coverage set of miRNA-target predicted interactions, which consist of pairs predicted by at least one prediction method with high and moderate confidence, respectively. We also collected experimentally validated miRNA targets in human from a recent release of TarBase [21]. To study the effects of having validated interactions in these networks on our analyses, for both the high-confidence and high-coverage networks, we further considered either having only the computational predictions, or both the computational predictions and experimentally validated pairs combined, resulting in 4 integrated miRNA-target networks in total (Table 1).Each of these networks can be represented either by a binary matrix or a bipartite graph (Figure 1). In the matrix representation, each row corresponds to an mRNA and each column corresponds to a miRNA. An element has value 1 if the miRNA represented by the column targets the mRNA represented by the row in the network, and 0 otherwise. In the graphical representation, each node in the first part represents an mRNA and each node in the second part represents a miRNA. There is an edge connecting a miRNA node and an mRNA node if the miRNA targets the mRNA.An idealized definition of an MTB is a set of miRNAs and mRNAs in which each of these miRNAs targets all these mRNAs but not any other mRNAs, and each of these mRNAs are targeted by all these miRNAs not any other miRNAs (Figure 1, type R). In the matrix representation, it corresponds to a submatrix (i.e., a subset of rows and columns not necessarily adjacent to each other) containing only 1’s, with all other elements on these rows and columns having value 0. In the graphical representation, it is a biclique (fully-connected bipartite subgraph) with no extra edges incident on these nodes. If the miRNA-target network was free of false positive and false negative errors, MTBs of this type would be perfect cases for testing the ceRNA hypothesis since they represent totally autonomous modules isolated from the other parts of the network.In practice, however, such ideal modules rarely exist in miRNA-target networks. Even if they do exist, they may not be observed in our integrated networks due to possible false positives and false negatives in the networks. We thus defined a number of other MTB types with less stringent requirements, by allowing some missing 1’s in the submatrix and/or extra 1’s in other elements on the defining rows and columns. We first defined three other types that retain the restrictive (R) requirement that the submatrix should contain all 1’s (i.e., the miRNAs in an MTB should target all mRNAs in the MTB), but either only the defining columns are not allowed to have extra 1’s (i.e., only the miRNAs (mi) are restricted from having extra interactions), or only the defining rows are not allowed to have extra 1’s (i.e., only the mRNAs (m) are restricted from have extra interactions), or the general case (gen) that both are allowed. The corresponding MTB types are denoted as Rmi, Rm and Rgen, respectively. Analogously, we also defined four loose (L) types that allow 0’s in the submatrix (i.e., the miRNAs in an MTB are not required to target all mRNAs in the MTB), resulting in the L, Lmi, Lm and Lgen types (Figure 1). Having different types of MTB enabled us to control the impacts of false positives and false negatives in the input network, and the amount of crosstalk between MTBs.The different MTB types have drastically different numbers of possible occurrences in a network (Figure 1). For some types, there is at most a linear number of MTBs with respect to the number of mRNAs and miRNAs in the network (types R and L). For some other types, the maximum number of MTBs is exponential, but the number of maximal MTBs, i.e., MTBs not being submatrices of other MTBs, is linear (types Rmi, Rm, Lmi and Lm). Type Rgen could give an exponential number of maximal MTBs in theory, but in practice a tractable number is usually observed. Finally, type Lgen has an exponential number of MTBs, both in theory and in practice. Consequently, we developed a variety of algorithms to identify MTBs of the different types, from simple graph searching algorithms that can efficiently identify all MTBs of a certain type, to algorithms that only return a subset of MTBs with the highest scores based on the intra-MTB density of interactions.

**Table 1:** **Summary statistics of the different datasets used in our study**

		High confidence			High coverage		
Dataset	Type	miRNAs	mRNAs	Interactions	miRNAs	mRNAs	Interactions
TarBase [21]	Validated	202	2,315	9,569	202	2,315	9,569
miRanda [6]	Predicted	409	567	1,000	856	2,633	5,000
miRGen [68]	Predicted	503	37	1,041	828	103	5,230
PicTar [69]	Predicted	91	438	1,000	164	1,541	5,000
PITA [70]	Predicted	290	760	1,000	582	2,708	5,000
TargetScan [71]	Predicted	29	214	1,000	40	948	5,000
Union (without TarBase)	Integrated	926	1,818	4,983	1,505	6,063	24,553
Expressed union (without TarBase)	Integrated	163	448	701	240	2,034	4,337
Union (with TarBase)	Integrated	1,063	3,711	14,548	1,631	7,208	34,111
Expressed union (with TarBase)	Integrated	181	605	1,020	256	2,188	4,653

The integrated datasets involve data from all the prediction sets with or without the experimentally validated miRNA-target pairs. Among them, the expressed union sets were formed by considering only the expressed miRNAs and mRNAs in the corresponding union sets. We used these four expressed union sets (with or without TarBase, high confidence or high coverage) in our analyses.

### Expression of miRNAs and mRNAs in the same MTBs are significantly more anti-correlated than general miRNA-target pairs

**Figure 2:**
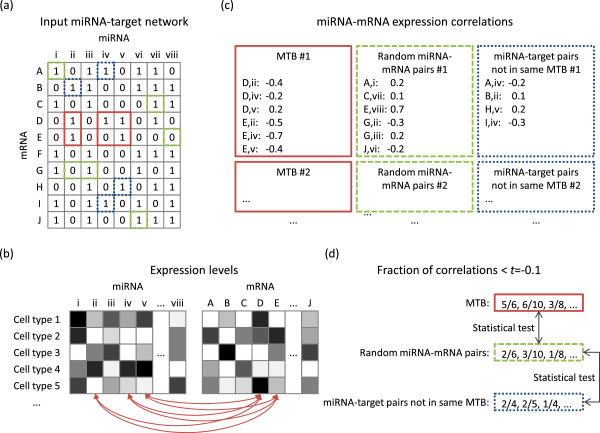
**Schematic figure explaining the workflow for testing the statistical significance of expression anti-correlation of miRNAs and mRNAs in the same MTBs. (a)** An example MTB (submatrix in cells with red borders), corresponding random miRNA-mRNA pairs (cells with green borders) and other targets of the miRNAs that define the MTB (cells with blue borders). **(b)** The expression levels of the miRNAs and mRNAs from multiple cell lines were collected. The expression correlation between each miRNA-mRNA pair in the MTB was computed. Similar correlation values were also computed for the two background sets (not shown). **(c)** For each MTB and corresponding background sets, the computed correlation values were recorded. **(d)** These correlation values were compared against a threshold *t* (-0.1 for example), and the fraction of correlation values more negative than *t* was computed. The vector of these fractional values from the MTBs was then compared to the vectors from the two background sets by a statistical test.

As a way to check whether the MTBs we identified represent modules with biological relevance, we examined the expression levels of the miRNAs and mRNAs in human cell lines obtained from RNA-seq experiments performed by the ENCODE Project Consortium [23, 24]. The details of the analysis pipeline are given in Materials and Methods (see also Figure 2 and Additional file 1: Figure S1a). Briefly, for each miRNA-mRNA pair in an MTB, we calculated the Pearson correlation of their expression across the cell lines. For each MTB, we then counted the fraction of pairs having correlation values more negative than a certain threshold *t*, multiple values of which (from -0.1 to -0.7) were tested. A large fraction of pairs having expression correlations more negative than the threshold would indicate that the regulatory effects of the miRNAs on the mRNAs were sufficiently strong to be observed in the expression data. To make sure that the negative correlations were not obtained by random chance, we compared these fractions with the corresponding fractions in random sets of expressed miRNAs and mRNAs of the same sizes as the identified MTBs. A p-value was then computed to determine if the fractions from the MTBs were significantly higher than those from the random background.

**Figure 3:**
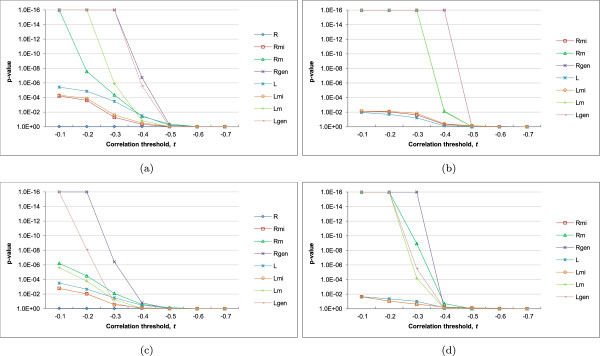
**Statistical significance of the negative correlations between the expression levels of miRNAs and mRNAs in the same MTBs.** The p-values were computed based on the expressed union sets with TarBase interactions, for **(a)** the high-confidence set and **(b)** the high-coverage set as compared to a random background sampled from all expressed mRNAs and miRNAs; and **(c)** the high-confidence set and **(d)** the high-coverage set as compared to a background consisting of miRNA-mRNA pairs with interactions in the input network but are not in same MTBs. In the figures, 1E-16 represents the smallest p-value that could be outputted by our program. MTB types with no identified MTBs are omitted.

In addition, we wanted to check if the negative correlations were simply a general phenomenon among miRNAs and their targets regardless of their MTB memberships. We therefore repeated the above procedure using a second background set of miRNA-mRNA pairs that composed of miRNAs and their targets not participated in the same MTBs.From the results for the expressed union set with TarBase interactions (Figure 3), we see that for moderate values of the correlation threshold (-0.1 to -0.4), for most MTB types, significantly more miRNA-mRNA pairs in the MTBs were anti-correlated in expression than random miRNA-mRNA pairs (panels a and b). For example, considering miRNA-mRNA pairs with expression correlation > -0.1, all MTB types except type R had a significantly higher fraction of such pairs than random miRNA-mRNA pairs at the p =0.01 level.Importantly, the miRNA-mRNA pairs in the MTBs were also significantly more anti-correlated in expression than miRNA-target pairs not in the same MTBs (Figure 3c,d), which suggests that the regulatory effects of miRNAs are either stronger or more clearly observed on their targets within the same MTBs than their other targets.

Significant p-values were obtained for both the MTBs from the high-confidence set (panels a and c) and the high-coverage set (panels b and d). We have also repeated our procedure for the networks without the validated miRNA-target interactions from TarBase (Additional file 1: Figure S2), and when related miRNAs with the same miRNA number but different modifiers (such as 5p and 3p) were grouped (Additional file 1: Figures S3 and S4). In all cases, the same general conclusion was drawn, that significantly more within-MTB miRNA-mRNA pairs were strongly anti-correlated in expression than random pairs and miRNA-target pairs not in the same MTBs. These consistent results show that MTB is a robust method for identifying miRNA-mRNA modules with strong expression relationships despite the fact that gene expression data were not used in defining the MTBs.

**Figure 4:**
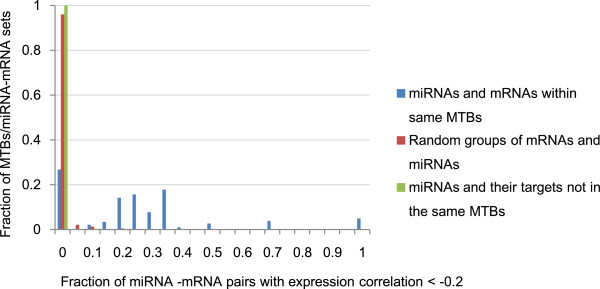
**Example fractions of miRNA-mRNA pairs satisfying the correlation threshold.** Type Lgen MTBs were identified from the high-confidence expression union set of miRNA-target interactions with TarBase inputs. For each MTB, the fraction of miRNA-mRNA pairs with expression correlation more negative than *t*=−0.2 among the 10 cell lines was computed. The distribution of these fractional values is shown by a histogram. Also shown are the distributions of fraction values for random groups of miRNAs and mRNAs of the same sizes as the MTBs, and for groups of miRNAs and their target mRNAs not within same MTBs.

Figure 4 shows the distributions of fractions of miRNA-mRNA pairs satisfying the correlation threshold in an example setting. It can be seen that for some MTBs, almost all miRNA-mRNA pairs (with a fraction close to 1) had expression correlations more negative than threshold *t*=−0.2. More generally, about two-third of the MTBs had more than 20% of their miRNA-mRNA pairs satisfying this correlation threshold. In contrast, for both random groups of miRNAs and mRNAs, and other miRNA-target pairs, almost none of them had expression correlation more negative than −0.2.

**Figure 5:**
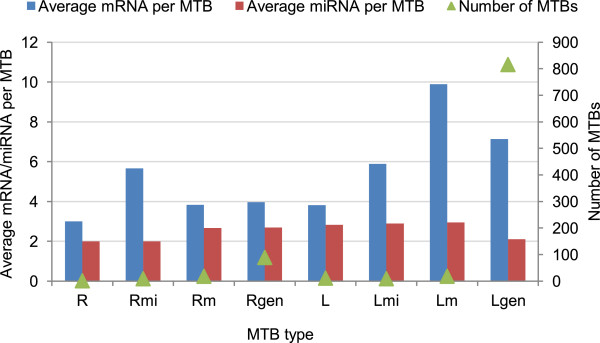
**Statistics of the MTBs identified from the high-confidence integrated expressed union set with TarBase interactions.** For each type of MTB, the average number of mRNAs per MTB, average number of miRNAs per MTB and the number of MTBs identified by our algorithm are shown.

Comparing the different MTB types, the general types that allow both the miRNAs to have extra-MTB targets and the mRNAs to be targeted by extra-MTB miRNAs (Rgen and Lgen) produced more MTBs as expected (Figures 5, Additional file 1: Figure S5–S7). Interestingly, the MTBs of these two types also contained miRNAs and mRNAs with more significant anti-correlations, and over a broader range of correlation threshold values (Figure 3). In contrast, due to the rigid requirements of type R, no MTBs of this type could be discovered from the high-coverage set and few were identified from the high-confidence set. Even when MTBs of this type could be found, their miRNA-mRNA anti-correlations of expression were not statistically significant. These results confirm the importance of explicitly considering non-fully-connected miRNA-mRNA modules and possible errors in the input miRNA-mRNA interaction networks.

#### Non-expression features can be used to identify MTBs with strong miRNA-mRNA expression anti-correlation

While the MTBs in general contained a significantly higher fraction of miRNAs and mRNAs with strong expression anti-correlation, we were interested in knowing whether some simple features of the MTBs could help identify the subset of MTBs with particularly strong expression anti-correlation without referencing the expression data. This would be particularly useful in identifying the most interesting MTBs when expression data are not available. To explore this possibility, for each MTB we identified, we computed 7 non-expression features, including the number of mRNAs and miRNAs in it, the density of 1’s in the MTB, in the same rows, columns or either but outside the MTB, and the MTB type. We then used these features to construct a Random Forest model [45] for predicting the fraction of miRNA-mRNA pairs within the MTB with expression correlation more negative than *t*=−0.1. Based on the results of 10-fold cross-validation, the average area under the receiver-operator characteristics (AUC) of ten equal-width fraction classes was 0.97, which is significantly higher than what would be expected for random predictions (AUC =0.5), indicating that the features were useful in identifying the MTBs with higher fractions of strong miRNA-mRNA expression anti-correlation.

We then looked for the features most important for identifying MTBs with strong expression anti-correlations between their member miRNAs and mRNAs. An exhaustive search of feature combinations identified two features that were consistently the most important in a 10-fold cross-validation procedure, namely the number of mRNAs in an MTB and the fraction of miRNAs outside an MTB that target the mRNAs in the MTB. Basically, MTBs with very strong anti-correlations between their miRNAs and mRNAs have a relatively small number of mRNAs and these mRNAs are targeted by few other miRNAs outside the MTBs, which are consistent with the intuition that MTBs with these properties are more autonomous, although the exact relationships of these two features with the fractions passing the correlation threshold are not linear in general.

#### Comparison with a previous method

To further check if MTBs represent novel miRNA-mRNA modules, we compared them with the miRNA regulatory modules (MRMs) identified by the Yoon and De Micheli method [46] from the same networks. It is one of the few methods in the literature that identify miRNA-mRNA modules from a miRNA-target network without requiring expression data as input. We applied the same procedure described above to check the fraction of miRNA-mRNA pairs within each MRM identified by this method with expression correlation more negative than a threshold. We then collected all these fractions, and compared them with the corresponding fractions from the MTBs. Since type Lgen was found to be most biologically relevant in terms of miRNA-mRNA expression anti-correlation, in this and subsequent analyses we focus on this type of MTBs.

**Figure 6:**
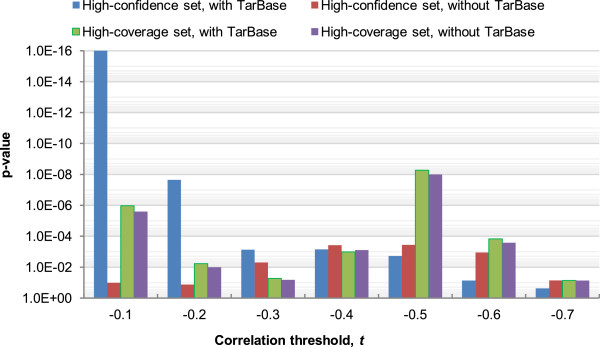
**Comparing MTBs with the miRNA regulatory modules (MRMs) identified by the Yoon and De Micheli method.** The p-values were computed by comparing the fractions of correlations more negative than the threshold *t* of miRNA-mRNA pairs within MTBs, as compared to those from the MRMs. In the figure, 1E-16 represents the smallest p-value that could be outputted by our program.

We found that for all threshold values between *t*=−0.1 and *t*=−0.7, there was constantly a higher fraction of miRNA-mRNA pairs within the MTBs passing the anti-correlation threshold than the MRMs identified by Yoon and De Micheli method as reflected by p-values > 0.5 (Figure 6). In many settings, the difference in these fraction values was statistically significant. For example, for all threshold values between *t*=−0.1 and *t*=−0.5, there was always a significantly higher fraction of miRNA-mRNA pairs in the MTBs passing the anti-correlation threshold than those in the MRMs at the *p*=0.01 level based on the high-confidence network with TarBase interactions. These results further confirm that the MTBs successfully identified groups of miRNAs and mRNAs with strong expression relationships from the miRNA-target networks alone.

### Potential widespread expression buffering between mRNAs in the same MTBs

After checking the biological relevance of MTBs, we then used them to study whether mRNAs commonly targeted by some miRNAs buffer each other in terms of their expression levels. We studied this question using three different methods.

First, we reasoned that if different mRNAs buffer each other, they should exhibit a positive correlation of expression levels across different cell types. To test if it was the case, we applied a procedure similar to the one we used for testing miRNA-mRNA anti-correlations described above. Specifically, we asked whether a significantly higher fraction of mRNA pairs in the same MTBs had expression correlation more positive than a threshold *t*, as compared to random mRNA pairs and mRNA pairs targeted by the same miRNA but not in the same MTBs.

**Figure 7:**
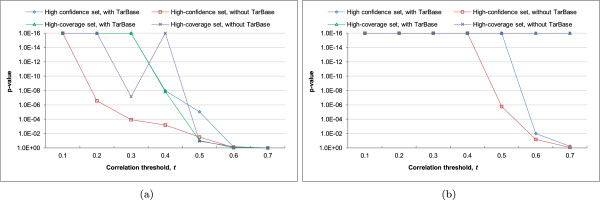
**Statistical significance of the positive Pearson correlations between the expression levels of mRNAs in the same type Lgen MTBs.** The p-values were computed by comparing the fractions of correlations more positive than the threshold *t* of the mRNA pairs within MTBs, as compared to **(a)** a background consisting of random mRNA pairs, or **(b)** a background consisting of mRNA pairs targeted by a common miRNA in the input interaction network but not in same MTBs. In the figures, 1E-16 represents the smallest p-value that could be outputted by our program.

The results (Figure 7) show that indeed significantly more mRNA pairs within type Lgen MTBs were strongly correlated in expression than both types of background mRNA pairs at various values of *t* from 0.1 to 0.4, no matter we considered the high-confidence or high-coverage set of miRNA target predictions, and whether experimentally validated pairs from TarBase were included or not. The p-values in the comparison with mRNA pairs targeted by same miRNAs but not in same MTBs as background were particularly significant (Figure 7b), indicating that MTBs helped discover mRNAs with strong expression correlations that could be hard to observe if all targets of a miRNA were considered together as a group.

We noticed that the positive correlations observed between mRNAs in the same MTBs are necessary but not sufficient for showing that they buffer each other. Since most mRNAs in the same MTB are expected to be targeted by the same miRNAs, a plausible alternative explanation is that the positive correlations were simply due to independent regulation by the same miRNAs without a feedback mechanism for the mRNAs to affect the expression level of each other. We argue that this co-regulation mechanism cannot fully explain the significant positive correlations observed, because mRNAs targeted by same miRNAs but not in same MTBs were not as correlated in expression as those in the same MTBs. Also, one possible situation in which mRNAs cannot back-regulate their targeting miRNAs, and thus they cannot buffer other mRNA targets, is when the miRNAs have saturated expression levels across different cell types. This was also unlikely the case since we observed significant anti-correlations between miRNAs and their mRNA targets in the same MTBs.

Nonetheless, the above arguments cannot rule out the possibility that the main function of MTBs was to identify the more reliable miRNA-target pairs from the noisy interaction network, and thus co-regulation effects between mRNAs in the same MTBs were still stronger than other mRNAs targeted by the same miRNAs according to the network.

To more directly distinguish between co-regulation and buffering, we applied a second analysis method. The main idea is that if some mRNAs buffer each other, the expression level of one mRNA would provide some information for explaining the expression level of another mRNA, even when the expression level of the targeting miRNAs have already been considered. In other words, we wanted to test if one mRNA could help explain the expression level of another mRNA that could not be fully explained by the miRNA targets alone. This idea can be quantified by using partial correlation. Suppose *R*,*T*_1_ and *T*_2_ represent a miRNA regulator, target mRNA 1 and target mRNA 2, respectively. We define *f*(*R*,*T*_1_) as the correlation between *R* and *T*_1_, and *f*(*R*,*T*_1_|*T*_2_) as the expected correlation between *R* and *T*_1_ given the level of *T*_2_. The difference between them, *d*(*R*,*T*_1_,*T*_2_)=*f*(*R*,*T*_1_|*T*_2_)−*f*(*R*,*T*_1_) would be negative if *T*_2_ provides additional information for explaining the expression anti-correlation between *R* and *T*_1_, and it would be close to 0 if *T*_2_ provides no additional information, such as when *T*_1_ and *T*_2_ were independently regulated by *R*. A similar method based on conditional mutual information was previously used to identify sponge modulators in miRNA-target networks [14].

**Figure 8:**
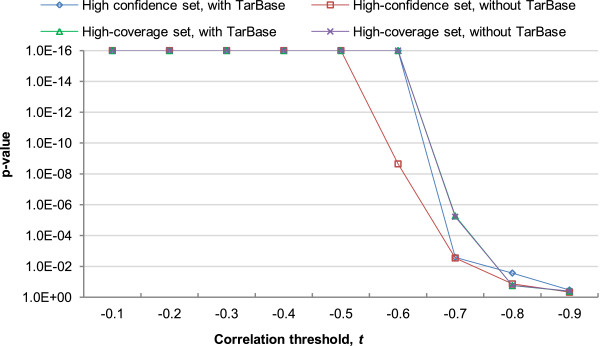
**Statistical significance of the extra information provided by an mRNA in our MTBs in explaining the relationship between a miRNA and another mRNA in the same MTB.** The p-values were computed by comparing the fractions of *d*(*R*,*T*1,*T*2) (see Materials and methods) more negative than the threshold *t* of the (*R*,*T*1,*T*2) combinations within MTBs, as compared to a background consisting of (*R*,*T*1,*T*2) combinations where *R* comes from the same MTBs but *T*1 and *T*2 do not. In the figure, 1E-16 represents the smallest p-value that could be outputted by our program.

Given *R* and *T*_1_ from an MTB, we compared the partial correlation values using other mRNAs from the same MTB as *T*_2_ with the values obtained by using other targets of *R* outside the MTB as *T*_2_. The results (Figure 8) show that as expected, significantly more mRNAs from the same MTBs gave a strong negative value of *d*(*R*,*T*_1_,*T*_2_) than other mRNA targets of the miRNAs, and the results were consistently obtained from all four miRNA-target networks. These results suggest that the mRNAs in an MTB do help explain the expression levels of each other in addition to what the regulating miRNAs can explain.

Finally, we reasoned that if two mRNAs buffer each other, they should have an expression correlation stronger than other mRNA pairs co-regulated by the same miRNA, even if we consider only those within the same MTBs. In other words, for any two mRNAs *T*_1_ and *T*_2_ from the same MTB, if *d*(*R*,*T*_1_,*T*_2_) is strongly negative, *f*(*T*_1_,*T*_2_) should be strongly positive. To test if this was the case, we picked the top *x* MTBs with most negative *d*(*R*,*T*_1_,*T*_2_) values and bottom *x* MTBs with most positive *d*(*R*,*T*_1_,*T*_2_) values. We then repeated the correlation analysis above (the first method) using either only the top MTBs or only the bottoms ones. For the 112 parameter settings we tested involving different input networks and different values of *x* and *t*, the top MTBs had equal or more significant p-values in 103 of the cases (92% of the 112 settings). This result confirmed our intuition that the top MTBs with potentially stronger expression buffering among its member mRNAs had their expression levels more correlated.

Taken together, the results of the three methods show that the mRNAs in the same MTBs are significantly correlated in expression, and this cannot be explained purely by the fact that they are regulated by the same miRNAs. We propose that one likely alternative explanation is that these mRNAs buffer each other in terms of their expression levels.

### mRNAs in same MTBs have related biological functions

In addition to expression correlations, another way to check the biological relevance of MTBs is to test whether the genes (mRNAs) in the same MTB are enriched in particular functional categories. We collected the Gene Ontology (GO) [47] annotation of the genes in each MTB, and computed the enrichment score of each GO term using both hypergeometric tests and EASE scores [48]. We then collected the most significant enrichment score of each MTB to form a distribution, and compared it with the corresponding distribution of a background set of mRNAs, where the background was either random sets of mRNAs with the same sizes as the MTBs, or mRNAs targeted by same miRNAs but not included in the same MTBs.

**Figure 9:**
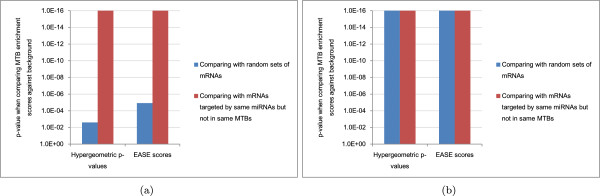
**Statistical significance of the functional enrichment scores of the genes from same type Lgen MTBs.** The p-values were computed based on the expressed union sets with TarBase interactions, for **(a)** the high-confidence set and **(b)** the high-coverage set. In the figures, 1E-16 represents the smallest p-value that could be outputted by our program.

From the results (Figure 9 and Additional file 1: Figure S8), it is seen that the genes in the MTBs were indeed more functionally related than both types of background mRNA sets. The results were largely unaffected by the exact way to compute enrichment scores (hypergeometric test p-values or EASE scores), although the results based on MTBs obtained from the high-coverage set of miRNA-target interactions were more significant.

#### Functional enrichment of mRNAs in same MTBs is not only due to co-expression

**Figure 10:**
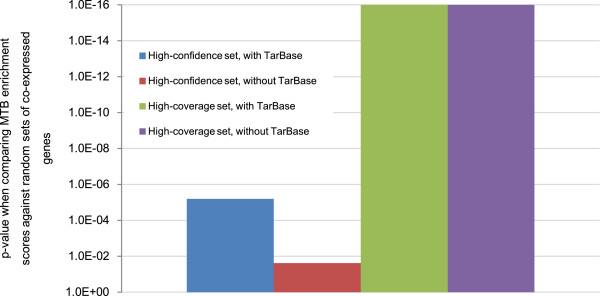
**Comparison of the functional enrichment scores of the mRNAs in same MTBs with random co-expressed mRNAs.** In the figure, 1E-16 represents the smallest p-value that could be outputted by our program.

Since the mRNAs in an MTB were correlated in expression in general, we further tested whether co-expression alone was sufficient to explain the functional enrichment. To test it, we sampled random sets of mRNAs with similar sizes and expression correlation profiles as the MTBs, and computed the hypergeometric test p-values of the GO terms of the mRNAs in each set. We then compared the distribution of the most significant enrichment score from each of these sets with the scores from the MTBs.The functional enrichment scores of the MTBs were found to be significantly stronger than the scores from the random sets of genes with similar levels of co-expression (Figure 10), especially when MTBs were identified from the two high-coverage miRNA-target networks. These results show that MTBs were able to identify groups of functionally related genes better than using co-expression information alone.

### MTB as a way to annotate miRNA functions

Finally, we explored the potential application of MTBs in identifying functionally related miRNAs. Currently, functional annotation of miRNAs is far less complete than protein-coding genes. Since each MTB represents a largely autonomous module, we hypothesized that the miRNAs in an MTB were functionally related to one another and to the mRNAs in the same MTB. To check if this was the case, for each MTB, we identified GO terms that were significantly enriched (p >0.05) based on the GO term annotations of the mRNAs. We then checked if these enriched GO terms were also related to the functions of the miRNAs in the same MTB. Table 2 shows several interesting examples we identified.

**Table 2:** **Illustrating examples of using MTBs to functionally annotate miRNAs**

Enriched GO term			
MTB ID	Total number of mRNAs	mRNAs annotated with the GO term	miRNAs
GO:0004674 Protein serine/threonine kinase activity			
1	4	AAK1, MAPK1, PDK3	miR-17, miR-20b, miR-93, miR-320a
GO:0000792 Heterochromatin			
2	4	CBX5, HMGA2, RNF20	let-7b, let-7c, let-7d, let-7e, let-7g, miR-185
GO:0007219 Notch signaling pathway			
3	3	CDK6, TNRC6B	miR-15a, miR-34a, miR-449a, miR-497
GO:0043161 Proteasome-mediated ubiquitin-dependent			
protein catabolic process			
4	19	EDEM1, UBE2W, WWP2	miR-25, miR-32, miR-363

Each row corresponds to one example MTB.

In the first example (MTB 1), the mRNA encoding AAK1, MAPK1 and PDK3 were annotated with the GO term “protein serine-threonine kinase activity”. We found that several miRNAs in MTB 1 are able to target the activities of the MAPK family of serine-threonine protein kinases. miR-320a has been shown to directly target MAPK1 activity to control the expression of pro-inflammatory cytokines in patients with myasthenia gravis [49]. Other miRNAs in the same MTB, miR-17 [50, 51] and miR-20b [50] can both target the MAPK signaling cascades to regulate cell cycle phase transition [50] and keratinocyte differentiation [51]. In addition, miR-93 also directly modulates the activity of the protein serine-threonine kinase, LATS2 to control tumor angiogenesis and metastasis in human breast cancer cells [52].

In MTB 2, genes encoding CBX5, HMGA2 and RNF20 are annotated by the GO term “heterochromatin”. The expression of HMGA2, a non-histone protein with important roles in chromosomal architecture and oncogenic transformation, is directly targeted by the let-7 miRNA [53, 54]. Interestingly, HMGA2 also functions as a ceRNA of Tgfbr3 through the let-7 miRNA family that commonly targets them, resulting in the promotion of lung cancer progression [55]. Furthermore, genome-wide chromatin-binding analysis suggested that let-7 and miR-185 are heterochromatin-bound miRNAs that can associate with AGO2 in the nucleus of senescent cells to mediate transcriptional gene silencing of proliferation-promoting genes [56]. Together, results from previously published studies supported our MTB classification of miR-185 and let-7 as miRNAs important in heterochromatin-binding and/or chromatin-remodeling.

We next examined the functions of the miRNAs in MTB 3, the mRNAs of which are annotated by the GO term “Notch signaling pathway”. We found that miR-34a inhibits cell proliferation in part by directly targeting the expression of CDK6 [57], an important cell cycle regulator whose expression is dependent on the Notch signaling pathway in T cell development [58]. Likewise, the level of miR-497 has been shown to inversely correlate with CDK6 expression to regulate cell cycle progression [59]. Furthermore, both miR-34a and miR-449a have been shown to target the expression of Notch1 [60, 61], a member of the Notch family of receptors in human cancer cell lines. Two of the miRNAs in MTB 3 can also target ligands of the Notch receptors: miR-34a is known to directly target Delta-like 1 [62] whereas miR-15a targets the non-canonical notch ligand, Delta-like 1 homolog [63].

Finally, in MTB 4, the mRNA-associated GO term “proteasome-mediated ubiquitin-dependent protein catabolic process” is also functionally related to the three miRNAs in the MTB. miR-25 has been shown to directly target the E3 ubiquitin ligase, WWP2 [64] to control the reprogramming of somatic cells to induced pluripotent stem cells. miR-363 directly inhibits a ubiquitin-specific protease, USP28 to promote proteasome-mediated degradation of Myc in human hepatocellular carcinoma [65]. miR-93, which lies in the miR-106b-25 cluster, has been shown to target the expression of *β*-TRCP2, a component of the SCF ubiquitin ligase complex important in the ubiquitination and subsequent proteasomal degradation of target proteins [66].

Collectively, these examples demonstrate that enrichment analysis based on the annotation of GO terms to the mRNAs in an MTB could be used as a way to annotate the functions of miRNAs in the same MTB.

### Discussion

In this study, we have shown that mRNAs in the same MTBs have significant expression correlations that cannot be explained purely by the fact that they are regulated by the same miRNAs. We have used multiple methods to show the high possibility that these mRNAs buffer each other in terms of expression, which suggests that ceRNAs could play an important role in the regulation of many mRNAs. In order to fully test the generality of the ceRNA hypothesis, it is necessary to perform perturbation experiments to see how the alteration of the expression level of one mRNA could affect other mRNAs regulated by the same miRNAs. Without such experimental data, in this study we do not aim at completely proving or disproving the generality of the ceRNA hypotheses. Instead, we think the MTBs represent small miRNA-target modules that could be very useful in identifying candidate miRNAs and mRNAs of future experimental studies in testing hypotheses related to ceRNA.

The fact that more significant p-values were observed for the MTB types with higher error tolerance (such as Rgen and Lgen) suggests that analysis results could indeed be misled by the errors present in the networks, and that trading off the purity of modules with some error tolerance is a reasonable strategy to handle the current noisy miRNA-target networks. On the other hand, although the Rgen and Lgen types of MTBs had the most statistically significant results, there could also be interesting cases identified by the other types. For instance, type R MTBs theoretically represent fully autonomous modules with complete target sharing among its member miRNAs, which are ideal cases for studying the ceRNA hypothesis. When miRNA-target networks become more complete and accurate in the future, more statistically significant results may be obtained from this and the other types of MTB with more stringent definitions.

One aspect of MTBs that we have not yet explored in this study is their cell-type specificity. Since MTBs are defined purely based on the miRNA-target connections, the different miRNAs and mRNAs in an MTB may not be all expressed in the same cell types. It would be interesting to study whether different miRNAs in an MTB usually co-express in the same cell types and co-regulate the common mRNA targets, or express in different cell types and act as alternative regulators.

In this work, we have focused on the use of expression data from ENCODE, which include matched mRNA and miRNA expression data from the same cell lines. We have also tested mRNA-mRNA positive correlations within our identified MTBs using a larger data set originally obtained from more than 73,000 microarray experiments [67]. Statistically significant results were again observed, but within a narrower range of correlation threshold *t*. We will test the concept of MTBs using larger data sets in the future.

While we have defined eight different types of MTB, actually they can all be described by a general framework. A detailed discussion is given in the Additional file 1. Briefly, a general MTB can be defined as a submatrix with an associated score, which is a combination of (1) the missing 1’s in the MTB, (2) extra 1’s in other rows of the defining columns, and (3) extra 1’s in other columns of the defining rows. Each of the eight MTB types corresponds to a particular way to combine these three components. While this general model appears to be more of theoretical interests, it actually has a real application in helping define MTBs from weighted networks in which each miRNA-mRNA pair is given a weight that indicates how likely they interact. Using such a weighted network would provide more information for analysis and avoid defining arbitrary thresholds to form a binary network.

## Conclusion

In this study, we have introduced microRNA-target biclusters (MTBs) as a method to systematically identify largely autonomous modules purely from the connections in a noisy miRNA-target network. To cater for modules involving miRNAs that do not target all mRNAs in the module, and the presence of false positives and false negatives in the network, we have defined eight different types of MTB with different levels of autonomy and error tolerance. We have shown that for some MTB types, especially those with higher error tolerance, the identified modules are biologically relevant by having significant anti-correlations between their member miRNAs and mRNAs as compared to both random miRNA-mRNA pairs and miRNA-target pairs not in the same MTBs. We have checked the robustness of our method using different input networks (high confidence or high coverage, with or without experimentally validated interactions), different values of the correlation threshold *t* in computing p-values, and whether to pre-group related miRNAs. The results were consistent across a wide spectrum of parameter settings.

The identified MTBs have enabled us to study how the expression patterns of their member mRNAs are related, with relatively small influence from other miRNAs and mRNAs outside the MTBs. Using three different analysis methods, namely direct expression correlation among the mRNAs, gain of miRNA-mRNA anti-correlation information by conditioning on another mRNA, and separate correlation analyses of MTBs with the strongest and weakest information gain, we have shown that there is strong correlation between the expression levels of mRNAs in the same MTBs that can well be explained by expression buffering as stated in the ceRNA hypothesis. These results show that although the regulatory effects of miRNAs are only partially reflected by the expression levels of their target mRNAs, and mRNA expression is affected by other regulatory mechanisms, it is still possible to use transcript levels to study the effects of miRNAs by decomposing a complex and noisy network of miRNA-target interactions into small modules that can be analyzed individually.

In the long term, the methods proposed in this study should be extended to model the hierarchical relationships between different MTBs and incorporate other regulatory mechanisms, to provide a more complete picture of the complex interactions between various types of biological objects in gene regulatory networks.

## Materials and methods

### Construction of miRNA-target networks

We collected experimentally validated human miRNA-target pairs from TarBase (v6.0) [21], which contained one of the most comprehensive sets of validated miRNA-mRNA interactions. We considered only the experimental types that likely report direct miRNA-mRNA interactions, namely PCR, ReportGeneAssay and Sequencing.

In addition, we gathered computationally predicted human miRNA-mRNA interactions using 5 methods based on different prediction approaches, namely miRanda (Aug 2010) [6], miRGen (v2.0) [68], PicTar (Hg18) [69], PITA (v6) [70] and TargetScan (6.0) [71]. The dataset for miRanda used was the human predictions with “Good mirSVR score, Conserved miRNA”, downloaded from http://cbio.mskcc.org/microrna_data/human_predictions_S_C_aug2010.txt.gz. The miRGen data file was downloaded from http://diana.cslab.ece.ntua.gr/data/public/TF_GENEID_miRNA_sorted.txt. The dataset for PicTar was the PicTar2 predicted target genes with conservation at the mammals’ level based on RefSeq gene models and human hg18 reference assembly, downloaded from http://dorina1.mdc-berlin.de/rbp_browser/hg18.html, choosing all genes in database in option 1 and all mammals’ miRNAs in database in option 2. For PITA, the Human Top predictions of miRNA targets were downloaded from http://genie.weizmann.ac.il/pubs/mir07/catalogs/PITA_targets_hg18_0_0_TOP.tab.gz. For TargetScan, the data used were the predicted conserved targets, downloaded form http://www.targetscan.org/vert_61/vert_61_data_download/Predicted_Targets_Info.txt.zip.

The gene names in all data files were converted to official gene symbols using the lookup table in HGNC [72]. Records with unrecognized gene names were ignored.

A high-confidence interaction network was constructed by taking the union of the 1,000 highest-scoring predictions from each method (where the number for miRGen was slightly larger due to ties in prediction scores). A second network was constructed by adding to this network the experimentally validated interactions in TarBase. Similarly, two high-coverage interaction networks were constructed by taking the union of the 5,000 highest-scoring predictions from each method, one with TarBase interactions and one without.

### Expression data

To study the expression levels of miRNAs and mRNAs across different cell types, we collected RNA-seq data in whole cells of human cell lines from ENCODE [23, 24], namely A549, AGO4450, BJ, GM12878, H1-hESC, HeLa-S3, K562, MCF7, NHEK and SK-N-SH, which contained the largest number of non-zero expression values for our mRNAs and miRNAs among all the human cell lines with RNA-seq data available from ENCODE at the time of collection. We used long PolyA+ RNA data to compute expression levels of mRNAs, and short total RNA data for miRNAs. Expression levels were computed by the number of reads mapped to each gene per kilobase per million reads (RPKM). We combined values from multiple replicates of the same experiment by taking their average.

As our goal was to study expression relationships between miRNAs and mRNAs, we focused on the set of mRNAs and miRNAs with non-zero expression values in at least 8 of the 10 cell lines. Considering only these miRNAs and mRNAs, we obtained the four integrated networks used in our analyses, namely the high-confidence/high-coverage expressed union network with/without TarBase interactions (Table 1).

### Definitions of MTBs and identification algorithms

As described in the Results section, we defined eight MTB types that differ in whether missing 1’s are allowed in the defining submatrix, and whether extra 1’s are allowed in the defining rows and columns outside the MTB (Figure 1). Here we provide detailed definitions of the eight types, and describe the corresponding algorithms for identifying the MTBs of each type from a miRNA-target network. In our analyses, by default we considered only MTBs containing at least two mRNAs and at least two miRNAs. For the analysis of positive expression correlations between mRNA pairs, in order to avoid having only one correlation value per MTB, we further considered only MTBs with at least 3 mRNAs.

#### Type R

Type R is the most restrictive type that requires each participating miRNA to target all participating mRNAs but no other mRNAs, and each participating mRNA to be targeted by all participating miRNAs but no other miRNAs. In the matrix representation, an MTB of this type is a submatrix with all 1’s, and all other elements on the same rows and columns are 0’s. Since each row and each column can participate in at most one MTB, the total number of MTBs is at most *min*(|*R*|,|*C*|), where *R* and *C* are the sets of all rows (i.e., mRNAs) and all columns (i.e., miRNAs), respectively, and the notation |*X*| denotes the size of any set *X*.

We developed an algorithm to identify all MTBs of this type from a miRNA-target network in linear time. The basic idea is to use the columns with 1’s in a row as its signature, and group all rows with the same signature together with the help of a hash table. Similarly, we defined the signature of a column as the rows at which it has 1’s, and grouped all columns with the same signature together. For each group of rows, if the columns in its signature do not have 1’s at other rows, it forms an MTB with these columns. Otherwise, by the definition of type R MTB, the whole group of rows cannot be members of any MTB. In this algorithm, whether there are other 1’s in these columns can be efficiently checked by the following method. Suppose *r* is the group of rows, *c* is the set of columns defining its signature, and *j* is one of these columns. All columns in *c* do not have other 1’s if and only if *j* belongs to a group with signature *r* for all *j*∈*c*.

The pseudocode of the whole algorithm is given in Additional file 1: Algorithm 1.

#### Type Rmi

Type Rmi is the same as type R except that the mRNAs of an MTB are allowed to be targeted by additional miRNAs. In the matrix representation, extra 1’s are allowed in other columns of the defining rows. There can be an exponential number of type Rmi MTBs, because if (*r*,*c*) is an MTB, then (*r*,*c*^′^) is also an MTB for any set of columns *c*^′^⊂*c*. On the other hand, if we define a maximal MTB as one that is not a submatrix of another MTB, then each column can participate in at most one maximal MTB. Therefore the total number of maximal MTBs is at most |*C*|.

We modified the algorithm for type R to identify all maximal MTBs of type Rmi. For each column, we defined its signature as the rows at which it has 1’s. We then grouped all columns with the same signature with the help of a hash table. Each resulting group of columns and the rows in their signatures form a maximal type Rmi MTB, with no additional checking required.

The pseudocode of the algorithm is given in Additional file 1: Algorithm 2.

#### Type Rm

Type Rm is the transpose of type Rmi. It allows each miRNA of an MTB to target other mRNAs outside the MTB, but does not allow the mRNAs to have other targeting miRNAs. Using the same argument for type Rmi, there can be an exponential number of type Rm MTBs, but at most |*R*| maximal MTBs.

The algorithm we used for identifying all maximal type Rm MTBs is analogous to the one for type Rmi, except that we grouped rows based on their signatures instead.

The pseudocode of the algorithm is given in Additional file 1: Algorithm 3.

#### Type Rgen

Type Rgen maintains the requirement that all miRNAs participating in an MTB must target all participating mRNAs, but the miRNAs are allowed to have other targets and the mRNAs are allowed to be targeted by other miRNAs. This type of MTBs is best described by the graphical representation, where each MTB is a biclique, i.e., a complete subgraph with all the miRNA nodes connecting to all the mRNA nodes. Again, there can be an exponential number of MTBs, as each subgraph of a type Rgen MTB is also a type Rgen MTB. There can also be an exponential number of maximal type Rgen MTB. For example, if there are 2^|*C*|^−1 rows and the signature of each row is the same as its index, i.e., the first row has signature 000...001, the second row has signature 000...010, the third row has signature 000...011, and so on, then each of the 2^|*C*|^−1 non-empty column combinations participates in a different maximal MTB. Because of the exponential number of possible maximal MTBs, and the fact that finding maximal bicliques is NP hard [73], in theory it is infeasible to identify all maximal type Rgen MTBs in a miRNA-mRNA network.

In practice, however, both the number of maximal type Rgen MTBs and the size of each are small in the networks we studied. We therefore used an iterative algorithm to find all maximal type Rgen MTBs, based on the Apriori algorithm proposed for association rule mining [74, 75]. The basic idea is that if (*r*,*c*) is a type Rgen MTB, then for any *c*^′^⊂*c*, (*r*,*c*^′^) must also be a type Rgen MTB. One can then iteratively discover MTBs with two columns, three columns, and so on, by testing *k*-column sets in the *k*-th iteration, constructed by merging two (*k*−1)-column sets in the previous iteration.

The pseudocode of the algorithm is given in Additional file 1: Algorithm 4.

#### Type L

The definition of type L MTB involves three rules. First, each participating miRNA is allowed to target only some of the participating mRNAs, but it cannot target any other mRNAs. Second, each participating mRNA is allowed to be targeted by only some of the participating miRNAs, but it cannot be targeted by other miRNAs. Finally, in the graphical representation, the nodes that represent the participating rows and columns should all be connected, i.e., there should be a path between any two nodes. In other words, each type L MTB is a connected component. Since each row and each column can participate in at most one MTB, the total number of type L MTBs is at most *min*(|*R*|,|*C*|).

We used a standard breadth-first search algorithm to find all connected components, i.e., all type L MTBs, in linear time.

The pseudocode of the algorithm is given in Additional file 1: Algorithm 5.

#### Type Lmi

Type Lmi MTB differs from type L by allowing the participating mRNAs of an MTB to be targeted by additional miRNAs outside the MTB. Since each type Rmi MTB is also a type Lmi MTB, there is at maximum an exponential number of type Lmi MTBs in a miRNA-target network. Since each column can participate in at most one maximal MTB, there are no more than |*C*| maximal type Lmi MTBs.

It is easy to see that the set of maximal type Lmi MTBs is exactly the same as the set of maximal type L MTBs. The algorithm for finding all maximal type L MTBs can thus be used for finding all maximal type Lmi MTBs. However, we did not adopt this approach for two reasons. First, by doing so it would be meaningless to define type L and type Lmi MTBs as two separate types. Second, a maximal type Lmi MTB is likely to have many member miRNAs and mRNAs not having interactions, leading to a low density of interactions within the MTB.

We therefore developed an algorithm for finding high-scoring type Lmi MTBs instead. The score of an MTB is defined as the density of 1’s in the defining sub-matrix, where the density of 1’s in an MTB is defined as the number of 1’s divided by the total number of elements in the submatrix. The algorithm starts with the set of maximal type Rmi MTBs, which all have an interaction density of one by definition. We then removed MTBs that are too similar to another one. After that, for each column not in any MTB, we tested if it was reasonable to add it and all rows with a 1 in that column to the MTB. If the resulting density of 1’s in the new MTB did not drop below a certain threshold (which we set to 0.3), we considered the addition of the column as reasonable. If there were multiple reasonable additions, we chose the one with the highest resulting density of 1’s and repeated the process. Otherwise, the current MTB was returned as one of the high-scoring type Lmi MTBs.

The pseudocode of the algorithm is given in Additional file 1: Algorithm 6.

#### Type Lm

Type Lm MTB is the transpose of type Lmi MTB. All the discussions about type Lmi MTBs can be applied to type Lm MTBs by swapping the rows and columns.

The pseudocode of the algorithm for finding all high-scoring type Lm MTBs is given in Additional file 1: Algorithm 7.

#### Type Lgen

Type Lgen has the most relaxed definition among the eight types, and is likely the most practical one. Each miRNA in a type Lgen MTB is allowed to target only some of the mRNAs in the MTB, and is allowed to target other mRNAs. Likewise, each mRNA is allowed to be targeted by only some of the miRNAs in the MTB, and is allowed to be targeted by other miRNAs. To avoid having completely unrelated miRNAs and mRNAs in the same MTB, we maintained the connectedness requirement from type L. Since type Rgen is a special case of type Lgen, there can also be an exponential number of type Lgen MTBs. On the other hand, the number of maximal MTBs is limited by the number of connected components in the network, which is at most *O*(*min*(|*R*|,|*C*|)).

Due to the exponential number of type Lgen MTBs, both theoretically and practically, it is infeasible to return all of them. On the other hand, it is also not meaningful to return all maximal MTBs, since they are usually very sparse and contain miRNAs and mRNAs that are only weakly connected. Therefore as in the cases of type Lmi and type Lm MTB, we adopted a different approach to return high-scoring MTBs, which are MTBs with a high density of 1’s within the defining submatrices. We developed an algorithm to find these high-scoring MTBs, based on some ideas from a previously method proposed for finding communities in partite networks [76]. First, we used the algorithm for type Rgen MTBs to find all maximal bicliques, and called each of them a bicluster. We then removed biclusters that are too similar to another one. After this step, we iteratively added extra rows or columns to each MTB in ways similar to the algorithms for type Lmi and type Lm MTBs, except that when a column/row was added to an MTB, it was not required to also add the rows/columns with 1’s in the adding column/row. For each bicluster, the best addition was kept. The process was repeated until no more mRNAs or miRNAs could be added without causing the density to drop below a threshold.

The pseudocode of the algorithm is given in Additional file 1: Algorithm 8.

### Workflow for expression correlation analyses

We used a unified workflow for studying the negative correlations between miRNAs and mRNAs in an MTB (Figure 2 and Additional file 1: Figure S1a). Each time we considered one of the four integrated miRNA-target interaction networks of expressed miRNAs and mRNAs as input (Table 1, High-confidence/high-coverage expressed union with/without TarBase interactions). MTBs of the different types were identified from the network using the algorithms described above. For each MTB, we calculated the Pearson correlation between the expression levels of each pair of participating miRNA and mRNA across the human cell lines. We then summarized all these correlations by computing the fraction of them more negative than a correlation threshold *t*, multiple values of which (-0.1 to -0.7 with a step size of 0.1) were tested. After collecting all these fractions from the MTBs of a particular type, we compared them with the fractions from two backgrounds. The first one involved 1,000 random sets of expressed miRNAs and mRNAs with sizes matching the size distribution of the actual MTBs. The second one involved the same miRNAs and their other targets not included in the same MTBs as them. To quantify the comparisons, we used Wilcoxon rank-sum test to calculate a one-sided p-value for each MTB type at each value of *t*. A significant p-value would mean the fractions from the MTBs were significantly higher than the set of fractions in comparison. As our goal was to compare the results in various parameter settings rather than emphasizing on the significance of one particular set of results, the reported p-values were not corrected for multiple hypothesis testing. We remark that if one was to use the concept of MTB to identify one set of reliable miRNA-target modules for downstream analyses, the statistical significance of such modules should be carefully corrected taking into account the number of hypothesis tests performed.

We also repeated the analysis when different miRNAs with the same miRNA numbers but different modifiers (such as has-mir-121a and hsa-mir-121b) were grouped together. The expression value of each group was defined as the average expression of the member miRNAs.

In the same way, we also tested the positive correlations between mRNAs in same MTBs, in which case we computed the fractions of pairs with expression correlation higher than a threshold *t*, where *t* took values from 0.1 to 0.7. The fractions obtained from mRNA pairs in same MTBs were first compared to fractions from random pairs of expressed mRNAs, and then to pairs of mRNAs targeted by the same miRNAs but were not in the same MTBs.

### Predicting MTBs with strong miRNA-mRNA expression anti-correlation

We developed a method for predicting MTBs with strong miRNA-mRNA expression anti-correlation when expression data are unavailable. For each MTB, we constructed seven non-expression features, namely 1) its number of mRNAs, 2) its number of miRNAs, 3) the density of 1’s in its submatrix, 4) the density of 1’s in other rows of the defining columns, 5) the density of 1’s in the other columns of the defining rows, 6) the density of 1’s in the other rows of the defining columns or the other columns of the defining rows, and 7) the MTB type. Each MTB was thus represented by a vector of seven numeric values. The goal was to identify the anti-correlation class of each MTB, where ten equal-width classes were defined based on the distribution of average anti-correlation values of the MTBs. We then took 9/10 of the MTBs to train a Random Forest model using the implementation in Weka [77], and tested its accuracy using the remaining 1/10 of MTBs with their anti-correlation classes hidden. We repeated the process with 10 random sets of training-testing data, and reported their average area of the receiver operator characteristics (AUC).

### Comparison with miRNA regulatory modules from Yoon and De Micheli

We compared the miRNA-mRNA expression anti-correlation with the miRNA regulatory modules (MRMs) from Yoon and De Micheli [46]. We implemented this method and applied it to find MRMs from each of our input miRNA-target networks. For each identified MRM, we computed the fraction of miRNA-mRNA pairs with expression correlation more negative than a threshold *t*. We then compared these fraction values with the fraction values from our type Lgen MTBs using a one-sided Wilcoxon rank-sum test. A significant p-value would indicate that the fraction values from the MTBs were significantly higher than the MRMs.

### Workflow for testing whether correlated expression of mRNAs were more likely due to buffering than co-regulation

To test if the correlated expression of two mRNAs in the same MTB is due to buffering or co-regulation, we applied a method similar to the one in Sumazin et al. [14]. The idea is to compute *d*(*R*,*T*_1_,*T*_2_)=*f*(*R*,*T*_1_|*T*_2_)−*f*(*R*,*T*_1_), where *R* is a regulating miRNA, *T*_1_ and *T*_2_ are two mRNA targets of it, *f* is the Pearson correlation function, and *f*(*R*,*T*_1_|*T*_2_) is defined as the expected correlation between *R* and *T*_1_ after dividing the cell lines into two groups based on the expression value of *T*_2_ (above mean and below mean). If *d*(*R*,*T*_1_,*T*_2_) is negative, it would mean that the expression relationship between *R* and *T*_1_ can be better explained when the expression of *T*_2_ is known, and thus *T*_1_ and *T*_2_ are not independently regulated by *R*, but they affect each other possibly due to buffering.

To globally test if the (*R*,*T*_1_,*T*_2_) combinations in our MTBs have significantly more negative *d*(*R*,*T*_1_,*T*_2_) values than combinations involving the same *R* but *T*_1_ and *T*_2_ outside our MTBs, we used a procedure similar to checking the anti-correlations between miRNAs and targets in MTBs, but with the distribution of anti-correlation values replaced by these *d*(*R*,*T*_1_,*T*_2_) values. The fraction of (*R*,*T*_1_,*T*_2_) combinations with a *d*(*R*,*T*_1_,*T*_2_) value more negative than a threshold *t* was computed for each MTB, and the resulting distribution of fractions from all MTBs was compared to the background distribution with the same *R*’s but *T*_1_’s and *T*_2_’s outside the MTBs using a one-sided Wilcoxon rank-sum test.

Based on the above calculations, we also collected *x* MTBs with the most negative *d*(*R*,*T*_1_,*T*_2_) values and the *x* with most positive *d*(*R*,*T*_1_,*T*_2_) values. We called the former set of MTBs the “top” MTBs and the latter set the “bottom” MTBs as the former set was expected to exhibit stronger expression buffering among the mRNAs in each of them. We then used our correlation pipeline to test if the mRNA-mRNA correlations were significantly stronger than other mRNA pairs targeted by the same miRNAs but were not in the same MTBs based on different values of the correlation threshold *t*. Considering the 4 input miRNA-target networks, 4 values of *x* (100, 200, 500 and 1000), and 7 values of *t* (0.1 to 0.7), we compared the p-values from the top MTBs and from the bottom MTBs under the 4×4×7=112 parameter settings.

### Workflow for functional enrichment analyses

We also setup a workflow for evaluating the functional relationships between the genes in same MTBs (Additional file 1: Figure S1b). For each MTB, we collected the terms associated with each gene (mRNA) defined in Gene Ontology [47]. For each term, we then computed a p-value using hypergeometric test, to indicate the enrichment of the term in this set of genes as compared to the background set of all genes. To ensure robustness of our results, we also computed EASE scores as defined on the DAVID Web site [48], which can be considered a more stringent version of the p-values. The most significant p-value from each MTB was then collected to form a distribution, and it was compared to the most significant p-values from random sets of mRNAs of the same sizes of the MTBs. This comparison was quantified by a one-sided Wilcoxon rank-sum test, where a significant p-value would indicate that the genes in the MTBs were more enriched in same functional terms than random gene sets.

We also repeated the same analysis for sets of random mRNAs with a similar size and a similar level of co-expression as the MTBs.

### Availability

The source code and compiled programs we used for our analyses are available at http://yiplab.cse.cuhk.edu.hk/MTB/.

## Electronic supplementary material

Additional file 1: **Supplementary materials.** This file contains supplementary methods and supplementary figures. (PDF 2 MB)
