# Supplementary material for: Systematic exploration of autonomous modules in noisy microRNA-target networks for testing the generality of the ceRNA hypothesis
Source: BMC Genomics. 2014 Dec 24;15(1):1178. doi: 10.1186/1471-2164-15-1178 (PMC4367885; doi:10.1186/1471-2164-15-1178)
Supplement: Supplementary file 1 — Additional file 1: Supplementary materials. This file contains supplementary methods and supplementary figures. (PDF 2 MB) [file 12864_2014_6917_MOESM1_ESM.pdf]

Supplementary materials

Supplementary methods

Pseudocode of the MTB identification algorithms

Algorithm 1 Algorithm for identifying all type R MTBs from a miRNA-target interaction network

```
function TypeRMTB(R, C, A)                ▷ R: the set of all row indices, each representing an mRNA
                                          ▷ C: the set of all column indices, each representing a miRNA
                                          ▷ A: the adjacency matrix of the miRNA-mRNA network
    Define MapR as a (bit string → row index set) hash map, initialized to an empty map
    for each i ∈ R do                                                    ▷ For each mRNA i
        MapR.put(A[i, .], i), where A[i, .] represents the i-th row of matrix A
            ▷ Use the miRNAs targeting i as the key to get the existing set or create a new set in MapR, then add i to this set
    end for
    Define MapC as a (bit string → column index set) hash map, initialized to an empty map
    for each j ∈ C do                                                    ▷ For each miRNA j
        MapC.put(A[., j], j), where A[., j] represents the j-th column of matrix A
            ▷ Use j's mRNA targets as the key to get the existing set or create a new set in MapC, then add j to this set
    end for
    Define MTBs as the list of MTBs, initialized to an empty list
    for each c ∈ MapR.keys do                ▷ For each key c of MapR, i.e., each group signature
        Define r as a bit vector corresponding to the row indices of MapR.get(c)
            ▷ r is the members of the group, where each mRNA in r is targeted by only and all of the miRNAs in c
        if r is a key of MapC and MapC.get(r) == c then
                                              ▷ The miRNAs in c also target only and all of the mRNAs in r
            MTBs.add((r, c))                  ▷ The mRNAs and the miRNAs form an MTB
        end if
    end for
    Return MTBs
end function
```

Algorithm 2 Algorithm for identifying all maximal type Rmi MTBs from a miRNA-target interaction network

function $\textsc{TypeRmiMTB}(R, C, A)$ ▷ R: the set of all row indices, each representing an mRNA
▷ C: the set of all column indices, each representing a miRNA
▷ A: the adjacency matrix of the miRNA-mRNA network
  Define Map as a (bit string $\rightarrow$ column index set) hash map, initialized to an empty map
  for each $j \in C$ **do** ▷ For each miRNA j
    Map.put($A[., j]$, j), where $A[., j]$ represents the j-th column of matrix A
    ▷ Use j's mRNA targets as the key to get the existing set or create a new set in Map, then add j to this set
  end for
  Define $MTBs$ as the list of MTBs, initialized to an empty list
  for each $r \in Map$.keys **do** ▷ For each key r of Map, i.e., each group signature
    $MTBs$.add((r, Map.get(r))) ▷ The group signature (mRNAs) and the group members (miRNAs) form an MTB
  end for
  Return $MTBs$
end function

Algorithm 3 Algorithm for identifying all maximal type Rm MTBs from a miRNA-target interaction network

function $\text{TypeRmMTB}(R, C, A)$ ▷ R: the set of all row indices, each representing an mRNA
▷ C: the set of all column indices, each representing a miRNA
▷ A: the adjacency matrix of the miRNA-mRNA network

Define Map as a (bit string $\rightarrow$ row index set) hash map, initialized to an empty map

for each $i \in R$ **do** ▷ For each mRNA i

Map.put($A[i, .]$, i), where $A[i, .]$ represents the i-th row of matrix A
▷ Use the miRNAs targeting i as the key to get the existing set or create a new set in Map, then add i to this set

end for

Define $MTBs$ as the list of MTBs, initialized to an empty list

for each $c \in Map$.keys **do** ▷ For each key c of Map, i.e., each group signature

$MTBs$.add((Map.get(c), c)) ▷ The group members (mRNAs) and the group signature (miRNAs) form an MTB

end for

Return $MTBs$

end function

Algorithm 4 Algorithm for identifying all maximal type Rgen MTBs from a miRNA-target interaction network

function TypeRgenMTB(R, C, A) ▷ R: the set of all row indices, each representing an mRNA
▷ C: the set of all column indices, each representing a miRNA
▷ A: the adjacency matrix of the miRNA-mRNA network

Preprocess A to group all rows with identical signatures together and all columns with identical signatures together, using hash maps as in the algorithms for type R. Define the resulting matrix with no identical rows or columns as A', and its rows and columns as R' and C'

Define $MTBs$, $MTBsCurr$ and $MTBsNext$ as the lists of all MTBs discovered, MTBs for the current iteration and MTBs for the next iteration, respectively, all initialized to an empty list

for each $j \in C'$ **do** ▷ For each column of the pre-processed adjacency matrix

$MTBsNext$.add(($A'[.,j]$, j)), where $A'[.,j]$ is the j-th column of A'
▷ (A'[.,j], j) is an MTB, but may or may not be maximal

end for

while $MTBNext$ is not empty **do** ▷ Beginning of the k-th iteration, where k starts with 1

$MTBs$.addall($MTBsNext$) ▷ Add all MTBs in $MTBsNext$ to $MTBs$

$MTBsCurr := MTBsNext$

$MTBsNext := \emptyset$ ▷ All MTBs newly discovered in the previous iteration are to be worked on in this iteration

for each pair of MTBs (r_1, c_1) and (r_2, c_2) in $MTBsCurr$, such that the $k-1$ smallest indexes of both sets are the same **do** ▷ Trying to merge these two MTBs to form a new MTB

if $r_1 \cup r_2 \neq \emptyset$ **then** ▷ The two MTBs have some common rows

Define $M = (r_1 \cup r_2, c_1 \cap c_2)$ as a new MTB

Remove any MTBs (r, c) in $MTBs$ where $r \in r_1 \cup r_2$ and $c \in c_1 \cap c_2$ ▷ Remove any non-maximal MTBs

$MTBsNext$.add(M)

end if

end for

end while

for each MTB in MTBs **do**

Replace any grouped rows and columns with the original row and column indices in A

end for

Return $MTBs$

end function

Algorithm 5 Algorithm for identifying all type L MTBs from a miRNA-target interaction network

function $\textsc{TypeLMTB}(R, C, A)$ ▷ R: the set of all row nodes, each representing an mRNA
▷ C: the set of all column nodes, each representing a miRNA
▷ A: the miRNA-mRNA network in a (node $\rightarrow$ neighbor set) hash map format
  Define $S := R \cup C$ as the set of row and column nodes not in any MTB yet
  Define $MTBs$ as the list of MTBs, initialized to an empty list
  while $S \neq \emptyset$ **do** ▷ While there are still row or column nodes not in any MTB
    Get any node x from S
    Define M as the nodes in the connected component of x, initialized to an empty set
    Define Q as the set of newly discovered nodes in the connected component of x, initialized to $\{x\}$
    while $Q \neq \emptyset$ **do** ▷ While there may still be undiscovered members of the connected component
      Define Q' as the set of nodes in the connected component to be discovered, initialized to an empty set
      for each node $y \in Q$ **do**
        $Q' := Q' \cup A$.get(y) ▷ Add all neighbors of y to Q'
      end for
      $M := M \cup Q$ ▷ Add all nodes discovered in the previous iterations to the MTB
      $Q := Q' - M$ ▷ Determine the set of newly discovered members of the connected component
    end while
    $MTBs$.add(M) ▷ Add M as a new MTB
    $S := S - M$ ▷ Redetermine the nodes not yet associated with any MTB
  end while
  Return $MTBs$
end function

Algorithm 6 Algorithm for identifying high-scoring type Lmi MTBs from a miRNA-target interaction network

function TypeLmiMTB(R, C, A, d, ρ) ▷ R: the set of all row indices, each representing an mRNA
▷ C: the set of all column indices, each representing a miRNA
▷ A: the adjacency matrix of the miRNA-mRNA network
▷ d: minimum distance between two initial MTBs both of which are to be kept
▷ ρ: minimum density of an MTB
Define $MTBs :=$ TypeRmiMTB(R, C, A) as the starting set of MTBs
for each pair of MTBs $M_1 = (r_1, c_1)$ and $M_2 = (r_2, c_2)$ in $MTBs$ **do**
▷ Remove initial MTBs that are too similar to some others
if $dist(M_1, M_2) < d$ **then** ▷ Remove the smaller one if the two initial MTBs are too similar, checked in the same way as in Algorithm 1 of Du et al., 2008
if $r_1 + c_1 < r_2 + c_2$ **then**
$MTBs := MTBs$ - M_1
else
$MTBs := MTBs$ - M_2
end if
end if
end for
Define R' as the set of mRNAs not participating in any MTBs in $MTBs$
while $R' \neq \emptyset$ **do** ▷ Try adding one of the rows not in any MTBs to one of the MTBs
Define den as the highest density of the resulting MTB after any of the additions, initialized to 0
for each $i \in R'$ **do**
Define js as the columns with 1's in $A[i, .]$, the i-th row of A
for each $M = (r, c) \in MTBs$ **do**
if Density($(r \cup \{i\}, c \cup js)$) $> den$ **then** $den :=$ Density($(r \cup \{i\}, c \cup js)$)
end if
end for
end for
if $den > \rho$ **then** ▷ Perform the addition only if the resulting density is higher than the threshold
Add the rows and columns to the MTB
Remove the rows from R'
if the resulting MTB is identical to another one in $MTBs$ **then**
Remove it from $MTBs$
end if
else
Break the while loop
end if
end while
Return $MTBs$
end function

Algorithm 7 Algorithm for identifying high-scoring type Lm MTBs from a miRNA-target interaction network

function TypeLmMTB(R, C, A, d, ρ) ▷ R: the set of all row indices, each representing an mRNA
▷ C: the set of all column indices, each representing a miRNA
▷ A: the adjacency matrix of the miRNA-mRNA network
▷ d: minimum distance between two initial MTBs both of which are to be kept
▷ ρ: minimum density of an MTB
    Define $MTBs$:= TypeRmMTB(R, C, A) as the starting set of MTBs
    for each pair of MTBs $M_1 = (r_1, c_1)$ and $M_2 = (r_2, c_2)$ in $MTBs$ **do**
▷ Remove initial MTBs that are too similar to some others
        if $dist(M_1, M_2) < d$ **then** ▷ Remove the smaller one if the two initial MTBs are too similar, checked in the same way as in Algorithm 1 of Du et al., 2008
            if $r_1 + c_1 < r_2 + c_2$ **then**
                $MTBs := MTBs$ - M_1
            else
                $MTBs := MTBs$ - M_2
            end if
        end if
    end for
    Define C' as the set of miRNAs not participating in any MTBs in $MTBs$
    while $C' \neq \emptyset$ **do** ▷ Try adding one of the columns not in any MTBs to one of the MTBs
        Define den as the highest density of the resulting MTB after any of the additions, initialized to 0
        for each $j \in C'$ **do**
            Define is as the rows with 1's in $A[., j]$, the j-th column of A
            for each $M = (r, c) \in MTBs$ **do**
                if Density($(r \cup is, c \cup \{j\})$) $> den$ **then** $den :=$ Density($(r \cup is, c \cup \{j\})$)
                end if
            end for
        end for
        if $den > \rho$ **then** ▷ Perform the addition only if the resulting density is higher than the threshold
            Add the rows and columns to the MTB
            Remove the columns from C'
            if the resulting MTB is identical to another one in $MTBs$ **then**
                Remove it from $MTBs$
            end if
        else
            Break the while loop
        end if
    end while
    Return $MTBs$
end function

Algorithm 8 Algorithm for identifying high-scoring type Lgen MTBs from a miRNA-target interaction network

function TypeLgenMTB(R, C, A, d, ρ) ▷ R: the set of all row indices, each representing an mRNA
▷ C: the set of all column indices, each representing a miRNA
▷ A: the adjacency matrix of the miRNA-mRNA network
▷ d: minimum distance between two initial MTBs both of which are to be kept
▷ ρ: minimum density of an MTB
  Define $MTBs$:= TypeRgenMTB(R, C, A) as the starting set of MTBs
  for each pair of MTBs $M_1 = (r_1, c_1)$ and $M_2 = (r_2, c_2)$ in $MTBs$ **do**
▷ Remove initial MTBs that are too similar to some others
    if $dist(M_1, M_2) < d$ **then** ▷ Remove the smaller one if the two initial MTBs are too similar, checked in the same way as in Algorithm 1 of Du et al., 2008
      if $r_1 + c_1 < r_2 + c_2$ **then**
        $MTBs := MTBs$ - M_1
      else
        $MTBs := MTBs$ - M_2
      end if
    end if
  end for
  Define R' as the set of mRNAs not participating in any MTBs in $MTBs$
  Define C' as the set of miRNAs not participating in any MTBs in $MTBs$
  while $R' \neq \emptyset$ or $C' \neq \emptyset$ **do** ▷ Try adding one of the rows not in any MTBs to one of the MTBs
    Define $denR$ as the highest density of the resulting MTB after any of the additions, initialized to 0
    for each $i \in R'$ **do**
      for each $M = (r, c) \in MTBs$ **do**
        if Density($(r \cup \{i\}, c)$) $> denR$ **then** $denR :=$ Density($(r \cup \{i\}, c)$)
        end if
      end for
    end for
    if $denR > \rho$ **then** ▷ Perform the addition only if the resulting density is higher than the threshold
      Add the row to the MTB
      Remove the row from R'
      if the resulting MTB is identical to another one in $MTBs$ **then**
        Remove it from $MTBs$
      end if
    end if
▷ Try adding one of the columns not in any MTBs to one of the MTBs
    Define $denC$ as the highest density of the resulting MTB after any of the additions, initialized to 0
    for each $j \in C'$ **do**
      for each $M = (r, c) \in MTBs$ **do**
        if Density($(r, c \cup \{j\})$) $> denC$ **then** $denC :=$ Density($(r, c \cup \{j\})$)
        end if
      end for
    end for
    if $denC > \rho$ **then** ▷ Perform the addition only if the resulting density is higher than the threshold
      Add the column to the MTB
      Remove the column from C'
      if the resulting MTB is identical to another one in $MTBs$ **then**
        Remove it from $MTBs$
      end if
    end if
    if $denR \leq \rho$ and $denC \leq \rho$ **then**
      Break the while loop
    end if
  end while
  Return $MTBs$
end function

A unified general model of MTB

It is possible to generalize all eight types of MTB by one single unified model. A general MTB is defined as a submatrix of the input matrix of miRNA-mRNA interactions. Each MTB is associated with a score indicating its proximity to the ideal case. Specifically, let R and C be respectively the sets of all rows (mRNAs) and all columns (miRNAs) in the input matrix, r and c be the rows and columns involved in an MTB, a_{ij} represents the element at row i and column j of the matrix, and k_0, k_1 and k_2 are parameters with non-negative values. The score of an MTB (r,c), $f(r,c)$, is defined by the following formula:

$$f(r,c) \quad = \quad 1 - k_0 \frac{\sum_{i \in r, j \in c}(1 - a_{ij})}{|r||c|} - k_1 \frac{\sum_{p \in (R-r), j \in c} a_{pj}}{|R-r||c|} - k_2 \frac{\sum_{i \in r, q \in (C-c)} a_{iq}}{|r||C-c|}$$

The scoring formula consists of four parts. The first part is the constant value 1, which represents the maximum score of an MTB. The other three parts are penalty terms for missing 1's in the MTB, extra 1's on the same columns outside the MTB, and extra 1's on the same rows outside the MTB, respectively. The three parameters determine which penalty terms to apply and their relative weights. For the eight MTB types defined, the algorithms we used to identify the corresponding MTBs can be considered as algorithms for finding top-scoring MTBs, defined as some specific instantiations of this general model with different sets of parameter values, as shown in Table S1.

Table S1 Relationships between the unified general model and the MTBs identified by our algorithms for the eight types of MTB. *: For the L, Lmi, Lm and Lgen types, we also have an additional connectedness requirement between the rows and columns.

MTB type	k_0	k_1	k_2
R	∞	∞	∞
Rmi	∞	∞	0
Rm	∞	0	∞
Rgen	∞	0	0
L	0*	∞	∞
Lmi	1*	∞	0
Lm	1*	0	∞
Lgen	1*	0	0

For type R MTB, having any missing 1's in a submatrix or extra 1's on the same rows or columns outside the submatrix would result in a negative infinity score, which disqualifies it as a valid MTB. For type Rmi, the penalty terms for

missing 1's in a submatrix and extra 1's on the same columns outside it still apply, but the penalty for extra 1's on the same rows outside the submatrix is waived. Similarly, for type Rm, extra 1's on the same rows are penalized, but extra 1's on the same columns are not. For type Rgen, both are not penalized and any submatrix having only 1's is qualified as a MTB. For type L, extra 1's on the same rows and columns outside a submatrix are penalized, but missing 1's within it is not. Similar arguments apply for types Lmi, Lm and Lgen, except that in these cases the within-MTB density of 1's is used to evaluate the quality of a MTB, but the allowed extra 1's on the same rows or columns are simply ignored.

In addition to the eight standard types, new types of MTB can be defined by using different combinations of values for the parameters k_0, k_1 and k_2. High-scoring MTBs can be found by heuristic optimization algorithms. In some special cases, as with some of the eight standard types, it is also possible to derive efficient algorithms to return all MTBs or all maximal MTBs.

The scoring formula can also be written in another way. Suppose now r is a binary vector with $|R|$ entries in total, where an entry is 1 if the corresponding row is a member of the MTB of interest and 0 if not. Similarly, we redefine c as the binary vector with $|C|$ entries in total, where an entry is 1 if the corresponding column is a member of the MTB and 0 if not. The scoring formula can then be written in terms of these vectors and the whole miRNA-mRNA interaction matrix A:

$$f(r,c) = 1 - k_0 \frac{r^t r c^t c - r^t A c}{r^t r c^t c} - k_1 \frac{(\mathbf{1}-r)^t A c}{(R-r)^t (R-r) c^t c} - k_2 \frac{r^t A(\mathbf{1}-c)}{r^t r (C-c)^t (C-c)},$$

where $\mathbf{1}$ represents the binary vector of all 1's (with a length depending on the context).

The main reason to write the scoring formula using the vector and matrix representations is that the general MTB model can then be extended to handle non-binary interaction matrices, in which each element a_{ij} takes on a continuous value between 0 and 1 that represents the confidence of the miRNA targeting the mRNA. Correspondingly, one may also allow fractional values in the r and c vectors to represent fussy MTB memberships. With these changes, a whole series of other

well-established optimization methods can be applied to identify MTBs of high scores.

Supplementary figures

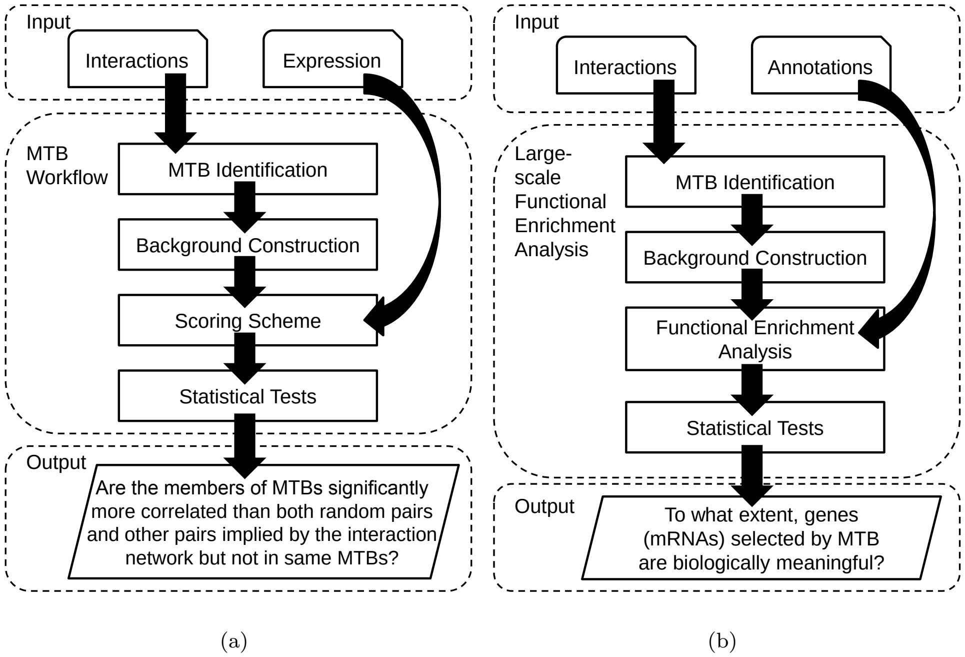

Figure S1 Workflow for studying (a) expression correlations between members of same MTBs and (b) functional relationships between genes in same MTBs.

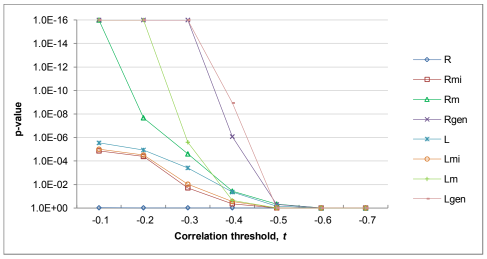

(a)

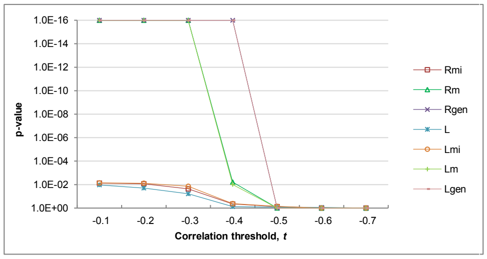

(b)

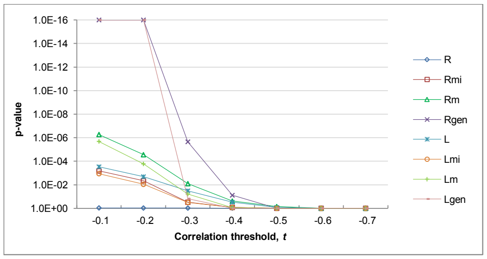

(c)

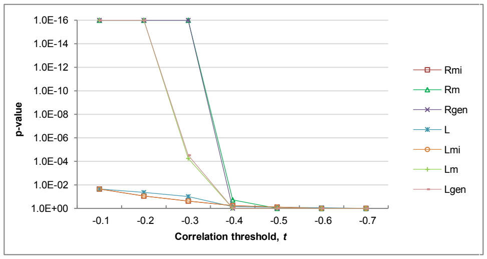

(d)

Figure S2 Statistical significance of the negative correlations between the expression levels of miRNAs and mRNAs in the same MTBs. The p-values were computed based on the expressed union sets without TarBase interactions, for (a) the high-confidence set and (b) the high-coverage set as compared to a random background sampled from all expressed mRNAs and miRNAs; and (c) the high-confidence set and (d) the high-coverage set as compared to a background consisting of miRNA-mRNA pairs with interactions in the input network but are not in same MTBs. In the figures, 1E-16 represents the smallest p-value that could be outputted by our program. MTB types with no identified MTBs are omitted.

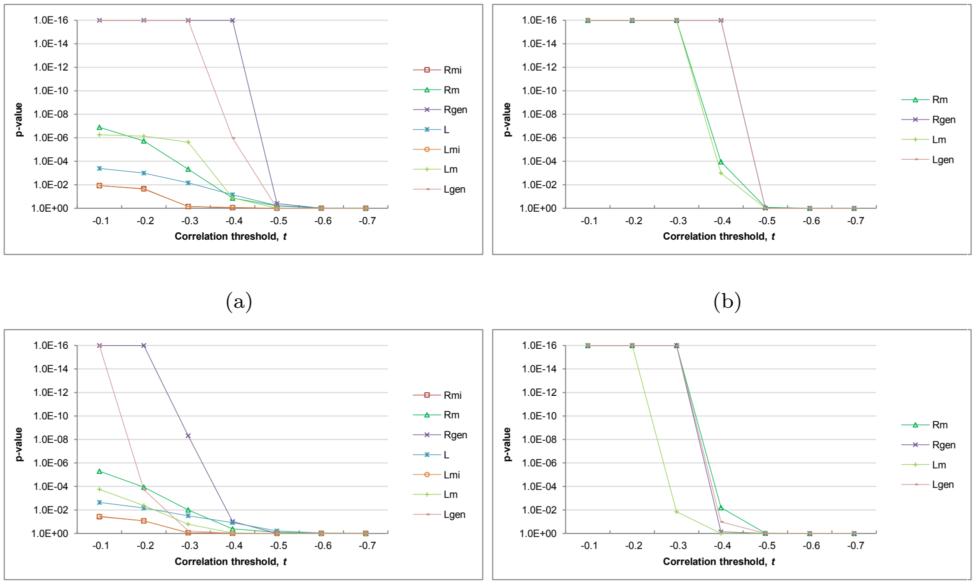

(a) (b)

(c) (d)

Figure S3 Statistical significance of the negative correlations between the expression levels of miRNAs and mRNAs in the same MTBs, when related miRNAs were grouped. The p-values were computed based on the expressed union sets with TarBase interactions, for (a) the high-confidence set and (b) the high-coverage set as compared to a random background sampled from all expressed mRNAs and miRNAs; and (c) the high-confidence set and (d) the high-coverage set as compared to a background consisting of miRNA-mRNA pairs with interactions in the input network but are not in same MTBs. In the figures, 1E-16 represents the smallest p-value that could be outputted by our program. MTB types with no identified MTBs are omitted.

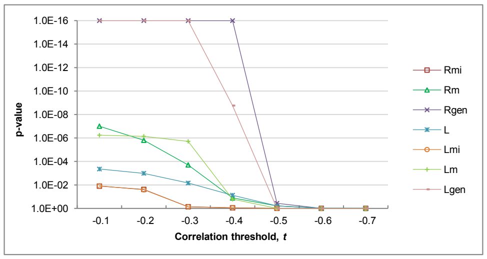

(a)

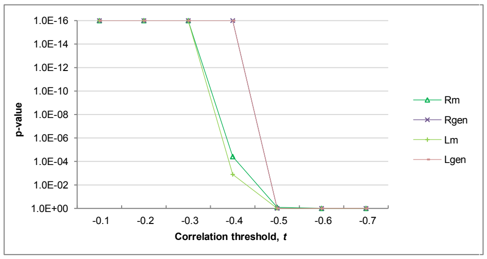

(b)

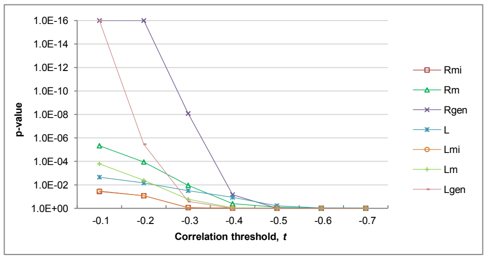

(c)

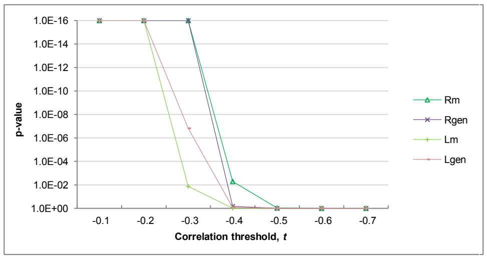

(d)

Figure S4 Statistical significance of the negative correlations between the expression levels of miRNAs and mRNAs in the same MTBs, when related miRNAs were grouped. The p-values were computed based on the expressed union sets without TarBase interactions, for (a) the high-confidence set and (b) the high-coverage set as compared to a random background sampled from all expressed mRNAs and miRNAs; and (c) the high-confidence set and (d) the high-coverage set as compared to a background consisting of miRNA-mRNA pairs with interactions in the input network but are not in same MTBs. In the figures, 1E-16 represents the smallest p-value that could be outputted by our program. MTB types with no identified MTBs are omitted.

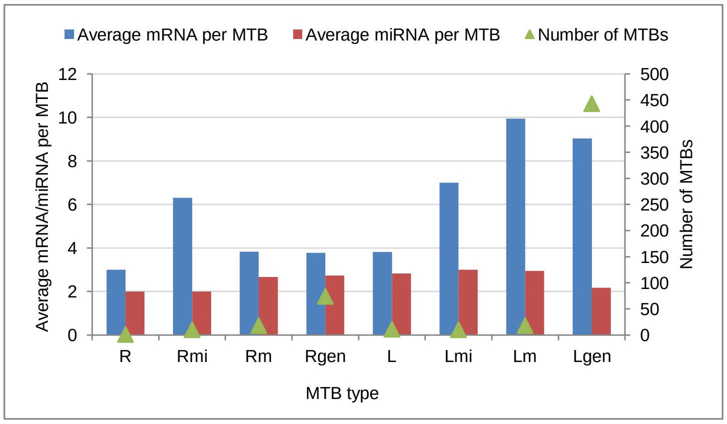

Figure S5 Statistics of the MTBs identified from the high-confidence expressed union set without TarBase interactions. For each type of MTB, the average number of mRNAs per MTB, average number of miRNAs per MTB and the number of MTBs identified by our algorithm are shown.

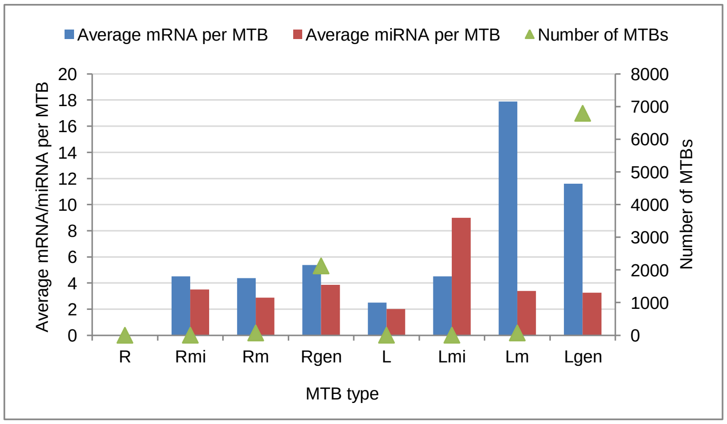

Figure S6 Statistics of the MTBs identified from the high-coverage integrated expressed union set with TarBase interactions. For each type of MTB, the average number of mRNAs per MTB, average number of miRNAs per MTB and the number of MTBs identified by our algorithm are shown.

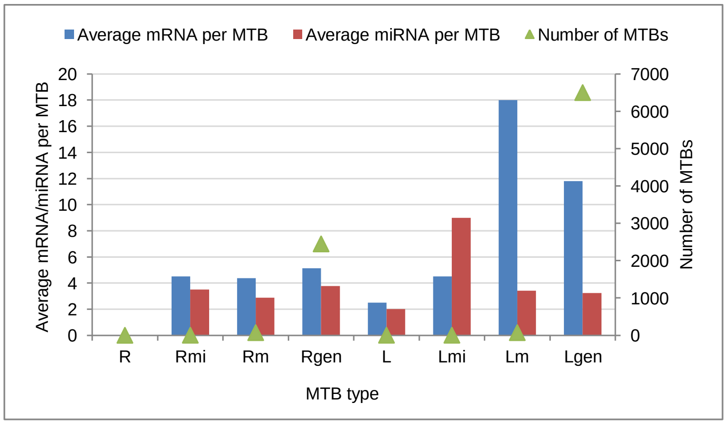

Figure S7 Statistics of the MTBs identified from the high-coverage integrated expressed union set without TarBase interactions. For each type of MTB, the average number of mRNAs per MTB, average number of miRNAs per MTB and the number of MTBs identified by our algorithm are shown.

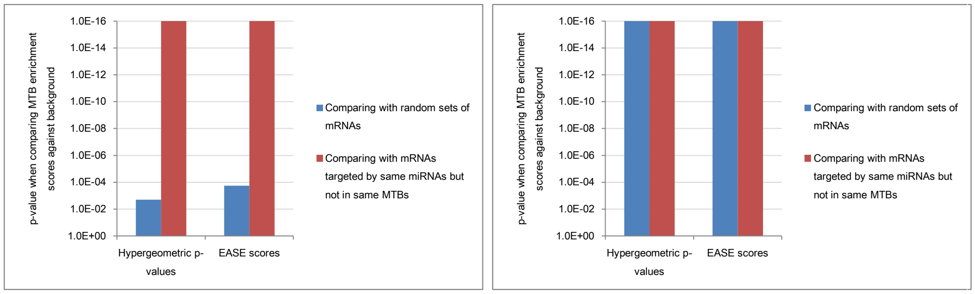

(a) (b)

Figure S8 Statistical significance of the functional enrichment scores of the genes from same type Lgen MTBs. The p-values were computed based on the expressed union sets without TarBase interactions, for (a) the high-confidence set and (b) the high-coverage set. In the figures, 1E-16 represents the smallest p-value that could be outputted by our program.
